# Diffractive Achromat with Freeform Slope for Broadband Imaging over a Long Focal Depth

**DOI:** 10.3390/mi14071401

**Published:** 2023-07-09

**Authors:** Donghui Yi, Fengbin Zhou, Jianyu Hua, Linsen Chen, Wen Qiao

**Affiliations:** 1 School of Optoelectronic Science and Engineering & Collaborative Innovation Center of Suzhou Nano Science and Technology, Soochow University, Suzhou 215006, China; suzhou08051012@163.com (D.Y.); 20214039002@stu.suda.edu.cn (F.Z.); jyhua@suda.edu.cn (J.H.); lschen@suda.edu.cn (L.C.); 2 Key Lab of Advanced Optical Manufacturing Technologies of Jiangsu Province & Key Lab of Modern Optical Technologies of Education Ministry of China, Soochow University, Suzhou 215006, China; 3 SVG Optronics, Co., Ltd., Suzhou 215026, China

**Keywords:** diffractive lens, achromatic, long focal depth, multi-level lens, micro-/nano-manufacture, focal efficiency

## Abstract

We propose a method for designing a long-focal-depth diffractive achromat (LFDA). By applying rotational symmetric parameterization, an LFDA with a diameter of 10.89 mm is designed over three wavelengths at six focal planes. The smoothly changed slope designed by the binary variable slope search (BVSS) algorithm greatly reduces the discontinuity in depth, thus it is a fabrication-friendly process for grayscale laser direct writing lithography, involving less fabrication error and cost. The deviation between the designed and fabricated profiles amounts to 9.68%. The LFDA operates at multiple wavelengths (654 nm, 545 nm, and 467 nm) with a DOF of 500 mm~7.65λ × 10^5^ (λ = 654 nm). The simulated and measured full-width at half-maximum (FWHM) of the focused beam is close to the diffraction limit. Experimental studies suggest that the LFDA possesses a superior capability to form high-quality chromatic images in a wide range of depths of field. The LFDA opens a new avenue to achieve compact achromatic systems for imaging, sensing, and 3D display.

## 1. Introduction

As a conventional optical element, the main function of a lens is to focus collimated light on a focal spot. The region where light is well focused in the direction of the optical axis is called the depth of focus (DOF). The numerical aperture (NA), the DOF, and the relationship between them define the beam properties [1]. Recently, there has been a lot of interest in extending the DOF, because it is beneficial not only for increased observation depths in imaging [2,3,4], but also for extended cutting depth in laser cutting and increased monitoring range in optical sensors. Moreover, a long focal depth is of great significance in glasses-free 3D display for crosstalk reduction and observation depth extension [5,6,7].

There are many methods to increase the DOF of a lens. In initial studies, axicons were adopted to enhance DOF, but the image resolution was severely degraded and the field of view was decreased [8,9]. Wavefront coding [10,11] and power-absorbing apodizers [12] are also used to extend the DOF. However, their applications are limited due to imaging quality degradation and increased device complexity [13]. Metasurface lenses employ a geometrical phase with nanorod structures to extend the DOF to 79λ [13]. Metalenses [14,15,16,17,18,19,20] have great advantages in manipulating the phase, amplitude, and polarization of light, yet large-scale fabrication difficulties for functional devices are detrimental to practical applications [21]. Properly designed diffractive lenses provide comparable, or even superior, optical performance to metalenses [22,23,24]. Furthermore, with larger feature size, diffractive lenses are accessible to low-cost, large-area and -volume manufacturing. The direct binary search algorithm [25,26,27] has been successfully adopted to design a diffractive lens with extended DOF at infrared wavelength [28], and the achromatic lens operates over the visible spectrum [29].

Here, we develop a binary variable slope search (BVSS) algorithm for the design of a long-focal-depth diffractive achromat (LFDA). By applying rotational symmetric parameterization, an LFDA with a diameter of 10.89 mm is designed over three wavelengths at six focal planes. The smoothly changed slope designed by the BVSS algorithm greatly reduces the discontinuity in depth of the LFDA. Grayscale laser direct writing lithography is adopted to fabricate the LFDA, involving less fabrication error and cost. The deviation between the designed and fabricated profiles amounts to 9.68%. The LFDA operates at multiple wavelengths with a DOF of 500 mm~7.65λ × 10^5^. The LFDA opens a new avenue to achieve compact achromatic systems for imaging, sensing, and 3D display.

## 2. Materials and Methods

### 2.1. Design

**Figure 1:**
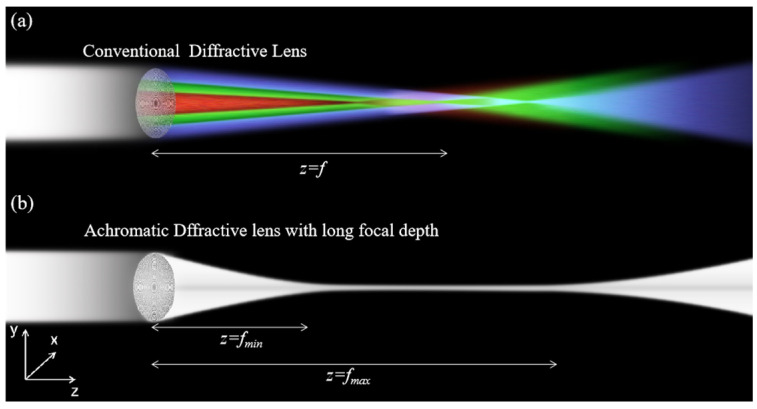
The schematic diagram of focused beam of (**a**) conventional diffractive lens and (**b**) LFDA.

From the microfabrication point of view, structures with sharp angles, straight side walls, or multiple steps generally involve extended fabrication time and increased fabrication error. Here, we propose LFDA with smoothly changed slope for extended DOF. Figure 1a is a conventional Frensel lens, in which the focal length significantly changes as a function of wavelengths. When illuminated by collimated white light, in comparison, the LFDA produces a focus beam for multiple wavelengths from *z* = fmin to *z* = fmax (Figure 1b), where *z* is the distance from the LFDA, and fmin and fmax are the minimum and maximum focal lengths, respectively.

The complex amplitude distribution *U*(*x*, *y*) of the designed diffractive lens on the image plane can be described as follows:

(1) $$U(x,y)=\frac{\exp(jkz)}{j\lambda z}\iint U_{0}(x_{0},y_{0})\exp\left\{\frac{jk}{2z}\left[{\left(x-x_{0}\right)}^{2}+{\left(y-y_{0}\right)}^{2}\right]\right\}dx_{0}dy_{0}$$

where *λ* is the incident wavelength, *k* equals *2π/λ*, (*x*, *y*) is the coordinates of the image plane, (*x*_0_, *y*_0_) is the coordinates of the incident plane, and *z* is the distance between the LFDA and the image plane.

The adopted evaluation function of BVSS algorithm is the average of the root-mean-square error (RWSE) of prescribed focus selected along the direction of the optical axis:

(2) $$\mathrm{RMSE}=\sqrt{\frac{\sum {\left(\left|F_{k}\right|-\left|F_{\mathrm{target}}\right|\right)}^{2}}{{\sum \left|F_{\mathrm{target}}\right|}^{2}}}$$

where *F_k_* is the reconstruction amplitude of the *k*th iteration, and *F_target_* is the target amplitude. 

The evaluation function is defined as

(3) $$R=\frac{1}{N}\sum_{i=1}^{N}a_{i}{\mathrm{RMSE}}_{i}$$

where *a_i_* is the weight factors.

**Figure 2:**
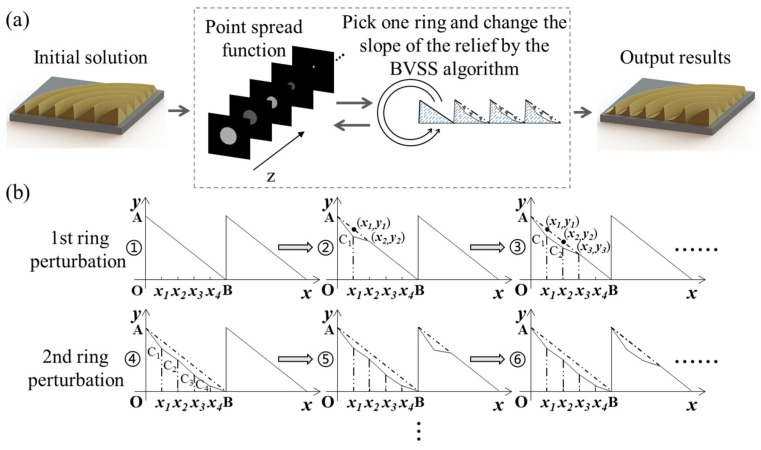
(**a**) The design flowchart of the LFDA. (**b**) The height perturbation process of a ring by BVSS algorithm.

**Figure 3:**
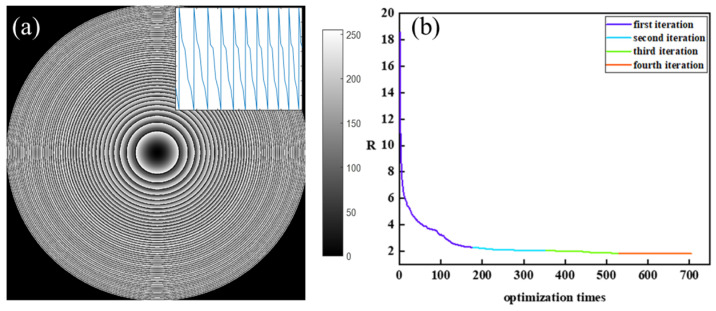
(**a**) The phase and cross-section profile of LFDA. (**b**) Evaluation function curve of BVSS algorithm.

Figure 2a shows the design flowchart of the LFDA. The parameterization of harmonic diffractive lens (HDL) with an appropriate aspect ratio is selected as a reference. The diffractive relief is calculated according to BVSS algorithm. Figure 2b is the height perturbation process of a ring by BVSS algorithm. Specifically, the surface profiling of each Fresnel zone from point A(0, *y*) to point B(*x*5, 0) is optimized by finding the position of C1, C2, C3, and C4. The ring is equally divided into five segments along *x* axis. Firstly, we randomly select point C1(*x*1, h1), where *x*1 is 1/5* × 5, h1 is a variable between 0 and *y*1. C1(*x*1, h1) is connected with A(0, *y*) and (*x*2, *y*2) to form two linear functions, whose slope varies with h1, as shown by ② in Figure 2b. An optimized h1 is searched between [0, *y*1] based on RMSE of multiple wavelengths at multiple prescribed focuses from Equation (2). Similarly, the optimized h2, h3, and h4 are searched in the range of [0, *y*2], [0, *y*3], and [0, *y*4], respectively. In this way, the height from A, C1, C2, C3, C4, to B monotonic decreases. The profiling of each Fresnel zone is successively optimized from center to the periphery, as shown in Figure 3a. The optimization proceeds with several iterations until R satisfies the termination condition. Figure 3b shows the corresponding evaluation function curves. The curves are divided into 4 segments labeled in four colors. Each segment corresponds to an optimization cycle in the algorithm step. The designed diffractive lens has 44 ringbands. Each cycle carries out 176 perturbations of height. Since the algorithm carries out 4 cycles, there are a total of 704 optimization calculations. The evaluation function value ends up being 1.79.

In contrast to the height perturbation of each pixel in the conventional binary search algorithm, the BVSS algorithm maintains the Fresnel zone architecture and optimizes the segmental slopes within each Fresnel zone. The BVSS algorithm reduces the amount of processing data and takes fabrication limitations into account.

Compared with multilevel DOE structures, a smooth surface profile is a much more fabrication-friendly microstructure for the grayscale laser direct writing process. In each Fresnel zone within a phase delay of 2π, the varied profiling within the zone contributes to an extended focal length. The dimensions of optimization parameters are shrunk to 1D by applying the rotational symmetric parameterization. Here, akin to the rotational symmetric design, LFDA with a diameter of 10.89 mm is designed over 3 wavelengths at 7 focal planes.

### 2.2. Simulation Results

In order to assess the efficiency of the proposed simulation method, we designed an LFDA at a central wavelength λ_0_ of 654 nm by scalar diffraction theory in the regime of Fresnel approximation. The harmonic diffraction coefficient is 5 and the harmonic wavelength is 545 nm and 467 nm. The radius of LFDA is 10.89 mm. The prescribed foci are designed at the distance of 50 mm, 100 mm, 125 mm, 166.6 mm, 250 mm, and 400 mm from the LFDA. The corresponding NA ranges from 0.0136 to 0.108. The maximum microrelief height is 5.45 µm. Positive photoresist AZ P4620 with a refractive index of 1.6 was used as the lens material both for simulation and fabrication.

We further analyzed the focusing efficiency of LFDA according to the following definition [30,31]:

(4) $$\eta =\frac{\sum I(p)}{\sum I},\forall p\in S$$

where *I* denotes the pixel values in the entire lens area, and symbol S represents the signal window which is the FWHM. The simulated averaged focusing efficiency is 38.58%, 43.29%, 40.95% for the wavelengths of 654 nm, 545 nm, and 467 nm, respectively. In comparison, the experimental average efficiency is 21.34%, 18.98%, and 20.78% of the LFDA at various positions for prescribed wavelengths, respectively (Figure S1 of Supplementary Materials). The fabrication errors contribute to the decreased focusing efficiencies. 

**Figure 4:**
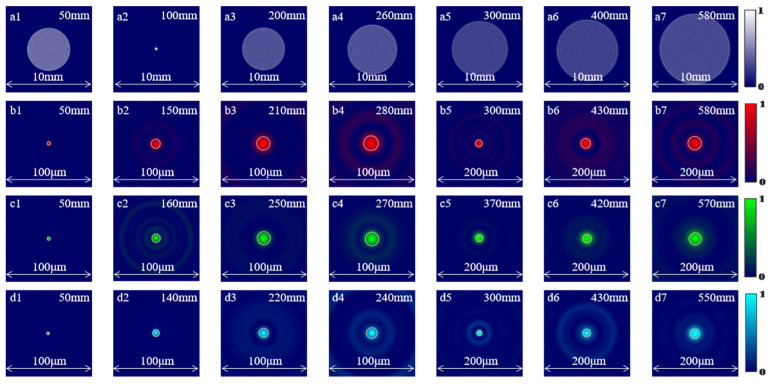
(**a1**–**a7**) The PSFs of the HDL at 654 nm in different axial positions. The PSFs of the LFDA at (**b1**–**b7**) *λ* = 654 nm, (**c1**–**c7**) *λ* = 545 nm, and (**d1**–**d7**) *λ* = 467 nm at different axial positions.

The simulated PSFs of the LFDA and the HDL at different incident wavelengths and different axial position are shown in Figure 4. The focal length of the HDL is 100 mm. The PSFs of the HDL at λ = 654 nm are diffused when moving away from the designed focal plane of 100 mm (Figure 4a1–a7). In contrast, the PSFs distribution of the LFDA remains in focus within a wide range of axial positions from 50 mm to 580 mm at λ = 654 nm (Figure 4b1–b7), from 50 mm to 570 mm at 545 nm (Figure 4c1–c7), and from 50 mm to 550 mm at 467 nm (Figure 4d1–d7). The circles in Figure 4 indicate the theoretical diffraction-limited FWHM.

## 3. Results

### 3.1. Fabrication of Long-Focal-Depth Achromatic Diffractive Lens

**Figure 5:**
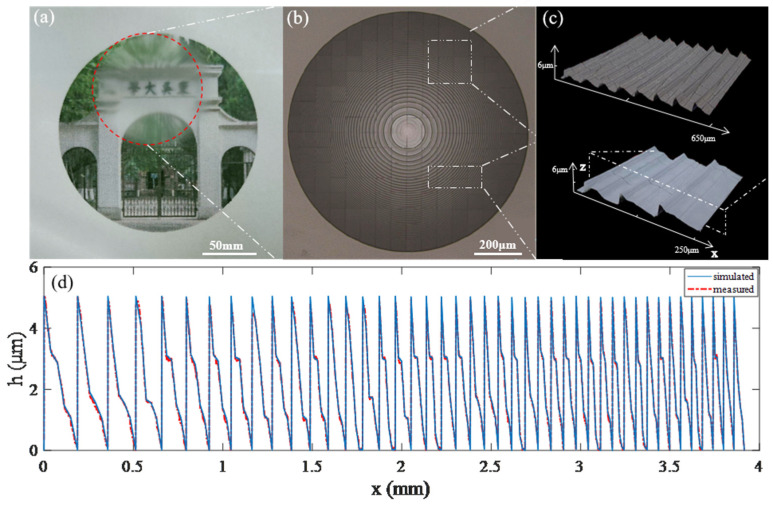
(**a**) The imaging result of LFDA. (**b**) Photograph of the fabricated LFDA. (**c**) Fragments of the LFDA. (**d**) Simulated (solid line) and measured (dashed line) profiles of the section of LFDA.

Based on the aforementioned simulation, an LFDA was fabricated by a homemade grayscale laser direct writing (GLDW) lithography system (MICROLAB, SVG Optronics, Suzhou, China). The detailed fabrication process has been described in prior studies [32,33,34,35,36]. Simply, a glass substrate was pre-cleaned and coated with a positive photoresist (AZ P4620, MicroChemicals, Baden Wuerttemberg, Germany) at a thickness of 6 µm. The glass substrate was then patterned with surface relief microstructures by GLDW technology. The LFDA sample, after development in 8‰ NaOH solvent, is shown in Figure 5a. The photograph of the LFDA and the 3D topography of two fragments of the LFDA are shown in Figure 5b,c, respectively. While the designed maximum depth is 5.45 µm, the height of the fabricated LFDA is 5.3 µm. The profile of the diffractive microrelief of the simulated (solid line) and fabricated LFDA (dashed line) is shown in Figure 4d. Standard deviation is defined by

(5) Standard deviation=∑i=1n(hi(measured)−hi(simulated)).^2/n

where *n* is the number of samples, and *h_i_*(*measured*) and *h_i_*(*simulated*) are the calculated and fabricated profiles, respectively. The standard deviation between the calculated and the fabricated profiles amounts to 9.68%, which makes it evident that the profile of the fabricated LFDA is in reasonable agreement with the required profile. This benefits from the fact that the microstructures we designed have fewer sharp angles and lower aspect ratios. 

### 3.2. The Point Spread Function of Long-Focal-Depth Achromatic Diffractive Lens

We summarize the evolution of PSFs along the *z* direction at the wavelength of *λ* = 654 nm, 545 nm, and 467 nm in Figure S2 (see Section S2, Supplementary Materials). The circles in Figure S2 also indicate the theoretical diffraction-limited FWHM. The FWHMs obtained from the measured PSFs are plotted as a function of *z* in Figure S3s–u (see Section S3, Supplementary Materials). The diffraction-limited and simulation-limited FWHMs are also drawn for comparison, and the simulated and measured FWHMs are close to the diffraction-limited FWHM for the different *z* at several prescribed wavelengths. Nevertheless, we show below that the LFAD is capable of forming reasonably good-quality images. 

The intensity distribution along the optical axis is measured at R/G/B wavelength. We estimate the DOF based on the FWHM [37] in the *z* direction. From Figure S4 in Supplementary Materials, the DOF of *λ* = 654 nm, *λ* = 545 nm, and *λ* = 467 nm is 530 mm (50 mm to 580 mm), 520 mm (50 mm to 570 mm), and 500 mm (50 mm to 550 mm), respectively. Thus, the DOF of the LFDA without chromatic aberration is 500 mm~7.65*λ* × 10^5^ (*λ* = 654 nm).

### 3.3. Characterization of the Long-Focal-Depth Achromatic Diffractive Lens

**Figure 6:**
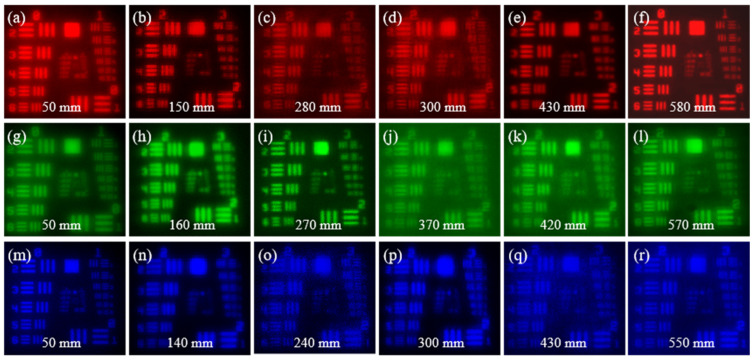
The images in different axial positions of resolution test chart of (**a**–**f**) λ = 654 nm, (**g**–**l**) λ = 545 nm, and (**m**–**r**) λ = 467 nm.

To evaluate the imaging performance of the LFDA, we used the resolution test chart (USAF 1951) as the object. A CCD sensor (AVT Prosilica GX2750C) with a pixel count of 2750 × 2200 and a pixel size of 4.54 µm was placed at different axial positions for image capturing. The LFDA forms relatively sharp images at the designed R/G/B wavelengths over a large focal depth (Figure 6). The averaged MTF at the contrast of 10% is 53.9 lp/mm over the distance from 50 mm to 580 mm for the wavelength of 654 nm. The averaged MTF at the contrast of 10% is 52.51 lp/mm over the distance from 50 mm to 570 mm for the wavelength of 545 nm. The averaged MTF at the contrast of 10% is 50.47 lp/mm over the distance from 50 mm to 550 mm for the wavelength of 467 nm (Figure S5 of Supplementary Materials). Computational imaging can be employed to further improve the optical performance [38,39,40].

**Figure 7:**
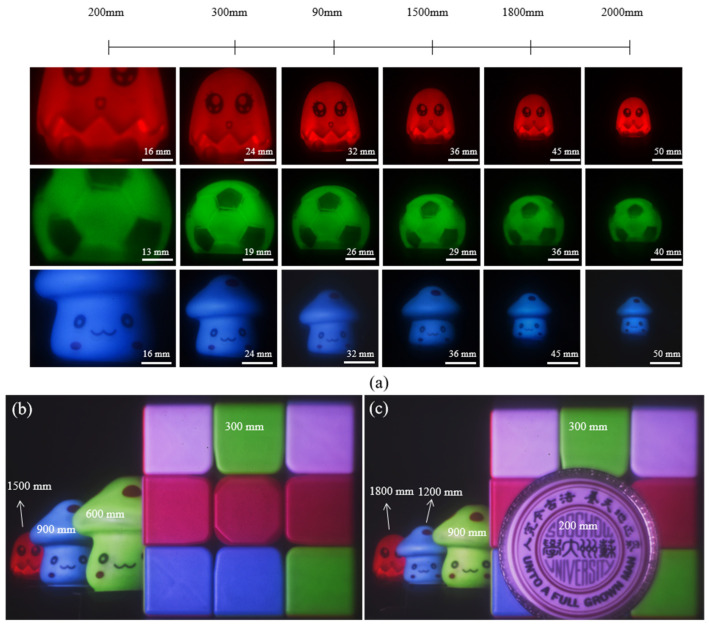
(**a**) The imaging results of red, green, and blue objects at different distances from the LFDA. (**b**,**c**) Imaging a scene with large depth of field with a camera consisting of only an LFDA and a conventional color CMOS sensor.

Figure 7a shows the imaging results for red, green, and blue objects at different distances from the LFDA. From geometric optics, magnification is a linear function of z. Finally, to evaluate the imaging potential of the LFDA, we imaged multiple objects that were placed over a wide distance range from 200 to 1800 mm (Figure 7b,c). Here, a single diffractive lens possesses a superior capability to form high-quality color images in a wide range of depths of field. 

## 4. Conclusions

In conclusion, we proposed a method to design an LFDA with a large DOF at multiple wavelengths. The LFDA was optimized by the BVSS algorithm. By applying rotational symmetric parameterization, the LFDA with a diameter of 10.89 mm was designed over three wavelengths at six focal planes. With a smoothly changed surface profile, the fabrication process of the designed LFDA is user-friendly, involving less fabrication error and cost. The deviation between the designed and fabricated profiles amounts to 9.68%. The LFDA operated at *λ* = 654 nm, *λ* = 545 nm, and *λ* = 467 nm with a DOF of 500 mm~7.65*λ* × 10^5^ (*λ* = 654 nm). The experimental studies demonstrated that the LFDA provides a wide broadband spectrum response over an extended distance range from 200 to 1800 mm. The LFDA opens a new avenue to achieve compact achromatic imaging or sensing systems, and provides novel solutions for glasses-free 3D display with extended viewing depth.

## Data Availability

The paper and Supplementary Information provide the data that support the results of our study. Additional supporting data generated during the study can be obtained from the corresponding authors.

## Supplementary Materials

The following supporting information can be downloaded at: https://www.mdpi.com/article/10.3390/mi14071401/s1, Table S1: Summary of the reported work in comparison to previously reported thin flat lens; Figure S1: The simulated focusing efficiency and the experimental measuring focusing efficiency of (a) 654 nm, (b) 545 nm, and (c) 467 nm; Figure S2: The PSFs of (a–f) λ = 654 nm, (g–l) λ = 545 nm, and (m–r) λ = 467 nm at different axial positions; Figure S3: Measured, simulated, and diffraction-limited full-width at half-maximum (FWHM) as a function of z for (s) λ = 654 nm, (t) λ = 545 nm, and (u) λ = 467 nm; Figure S4: The intensity profile of the PSFs along the axis for (a) λ = 654 nm, (b) λ = 545 nm, and (c) λ = 467 nm; Figure S5: The modulation transfer function for the LFDA at (a) λ = 654 nm, (b) λ = 545 nm, and (c) λ = 467 nm; Figure S6: The resolution chart measured at the wavelength of 654 nm; Figure S7: The resolution chart measured at the wavelength of 545 nm; Figure S8: The resolution chart measured at the wavelength of 467 nm; Figure S9: Experimental setup for imaging.
