# Supplementary material for: Diffractive Achromat with Freeform Slope for Broadband Imaging over a Long Focal Depth
Source: Micromachines (Basel). 2023 Jul 9;14(7):1401. doi: 10.3390/mi14071401 (PMC10383085; doi:10.3390/mi14071401)
Supplement: Supplementary file 1 [file micromachines-14-01401-s001.zip › micromachines-2467788-supplementary.pdf]

Supplementary Information

Diffractive achromat with freeform slope for broadband imaging over a long focal depth

Donghui Yi[1,2,†], Fengbin Zhou[1,2,†], Jianyu Hua[1,2], Wen Qiao[1,2,*], Linsen Chen[1,2,3]

1School of Optoelectronic Science and Engineering & Collaborative Innovation Center of Suzhou Nano Science and Technology, Soochow University, Suzhou 215006, China
2Key Lab of Advanced Optical Manufacturing Technologies of Jiangsu Province & Key Lab of Modern Optical Technologies of Education Ministry of China, Soochow University, Suzhou 215006, China
3SVG Optronics, Co., Ltd, Suzhou 215026, China
**Corresponding author. Email: wqiao@suda.edu.cn*
†These authors contributed equally to this work: Donghui Yi and Fengbin Zhou

This document provides supplementary information to "Diffractive achromat with freeform slope for broadband imaging over a long focal depth".

Comparison of Extended Depth of Focus Lenses

Table S1. Summary of the reported work in comparison to previously reported thin flat lens

	Achromatic characteristics	aperture	Depth of focus	Resolution	Ref.
LFDA (our work)	√	10.89 mm	50 mm-550 mm ($7.65\lambda\times10^5$, λ=654nm)	average MTF=53.9 lp/mm (contrast=0.1, λ=654nm)	
spectral diffractive lenses	√	8 mm	/	/	[1]
MDL	√	0.1 mm/0.37 mm	/	1-5[a] (NA=0.18)	[2]
	×	1.8 mm	5 mm-1200 mm ($10\lambda\times10^5$, λ=850nm)	average MTF=23 lp/mm (contrast=0.1)	[3]
Multiring pure-phase binary optical elements	×	7 mm	0.632 mm (594λ, λ=1064nm)	/	[4]
multiplexing programmable diffractive lenses	×	19.68 mm	900 mm-1100 mm ($4.37\lambda\times10^5$, λ=458nm)	4-6 (NA=51)	[5]

[a] Largest set of non-distinguishable lines of the Air Force resolution chart in different axial position.

1. The simulation and experimental focusing efficiency of the LFDA at several positions in the direction of the optical axis.

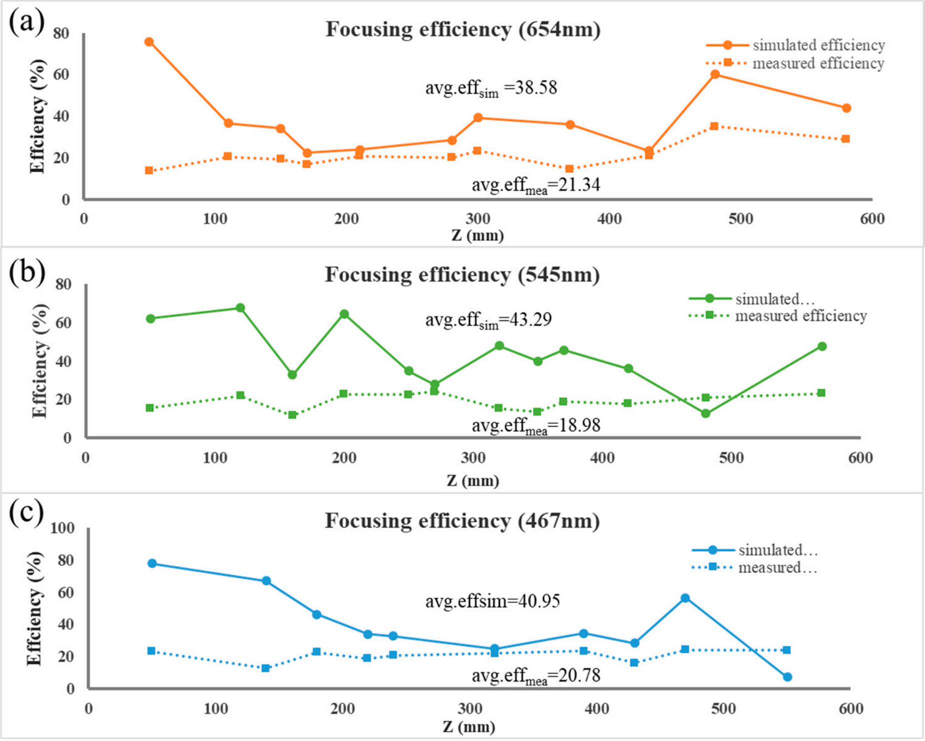

Figure S1. The simulated focusing efficiency and the experimental measuring focusing efficiency of the (a) 654 nm, (b) 545 nm, and (c) 467 nm.

LFDA is illuminated by a collimated mercury lamp. A CCD sensor is adopted to capture the focal stop of each wavelength. Next, the focusing efficiency was calculated using the following equation: Focusing efficiency = (sum of pixel values in $1 * \mathrm{FWHM}$ / sum of pixel values in the entire lens area).

2. The measured PSFs of the LFDA at different incident wavelengths and different axial position.

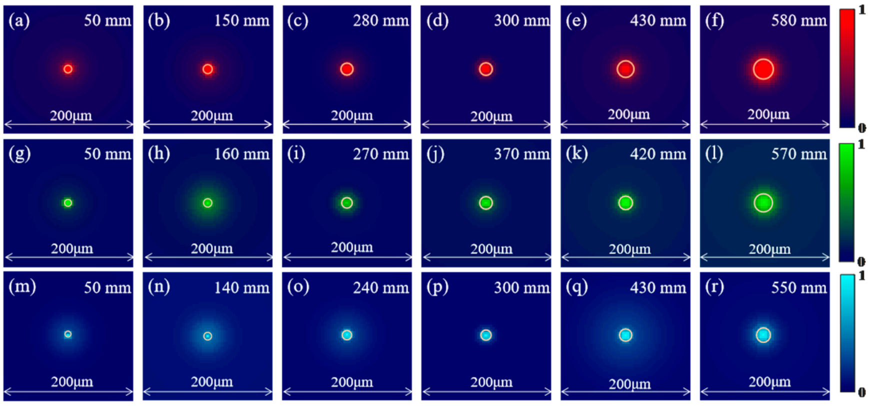

Figure S2. The PSFs of the (a)-(f) λ = 654 nm, (g)-(l) λ = 545 nm, and (m)-(r) λ = 467 nm at different axial position.

3. The FWHM obtained from the measured PSFs.

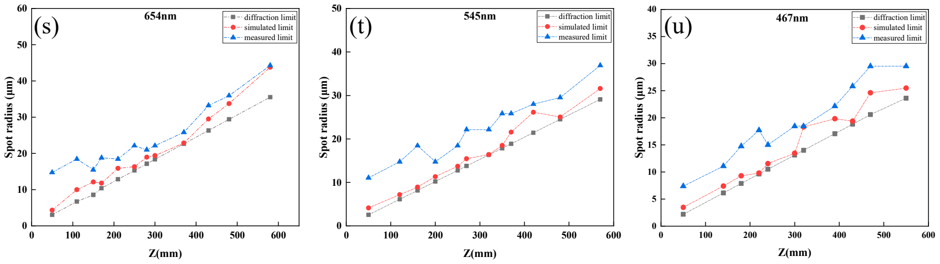

Figure S3. Measured, simulated and diffraction-limited full-width at half-maximum (FWHM) as a function of z for the (s) λ = 654 nm, (t) λ = 545 nm, and (u) λ = 467 nm.

4. The intensity distribution along the optical axis at R/G/B wavelength.

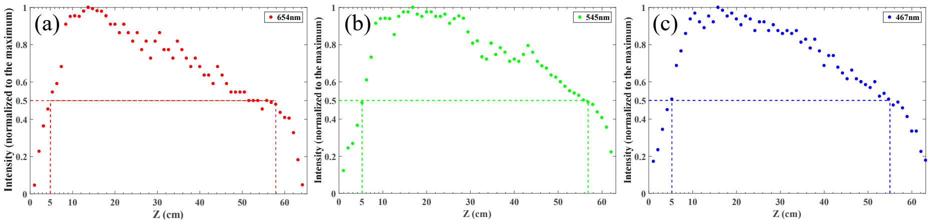

Figure S4. The intensity profile of the PSFs along the axis for (a) λ = 654nm, (b) λ = 545 nm, and (c) λ = 467 nm.

5. Modulation Transfer Function (MTF).

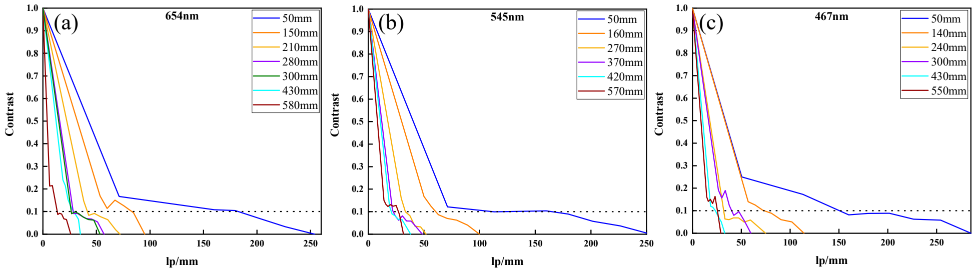

Figure S5. The modulation transfer function for the LFDA at (a) λ = 654nm, (b) λ = 545 nm, and (c) λ = 467 nm.

6. Imaging results at different focal planes for prescribed wavelengths using resolution test chart with filters.

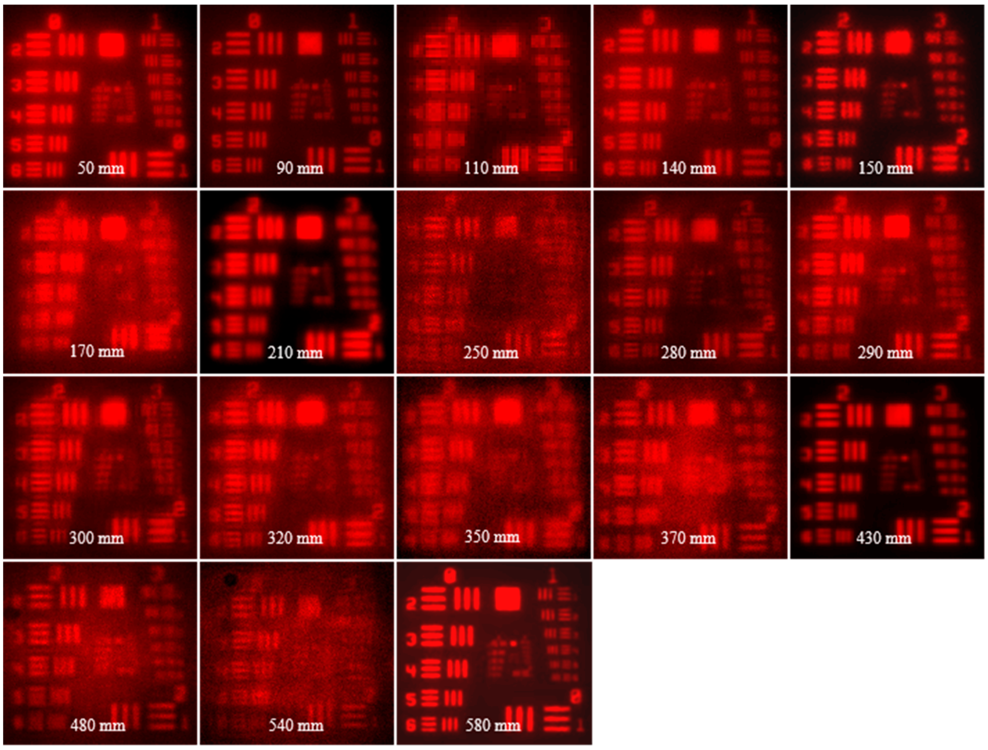

Figure S6. The resolution chart measured at the wavelength of 654 nm.

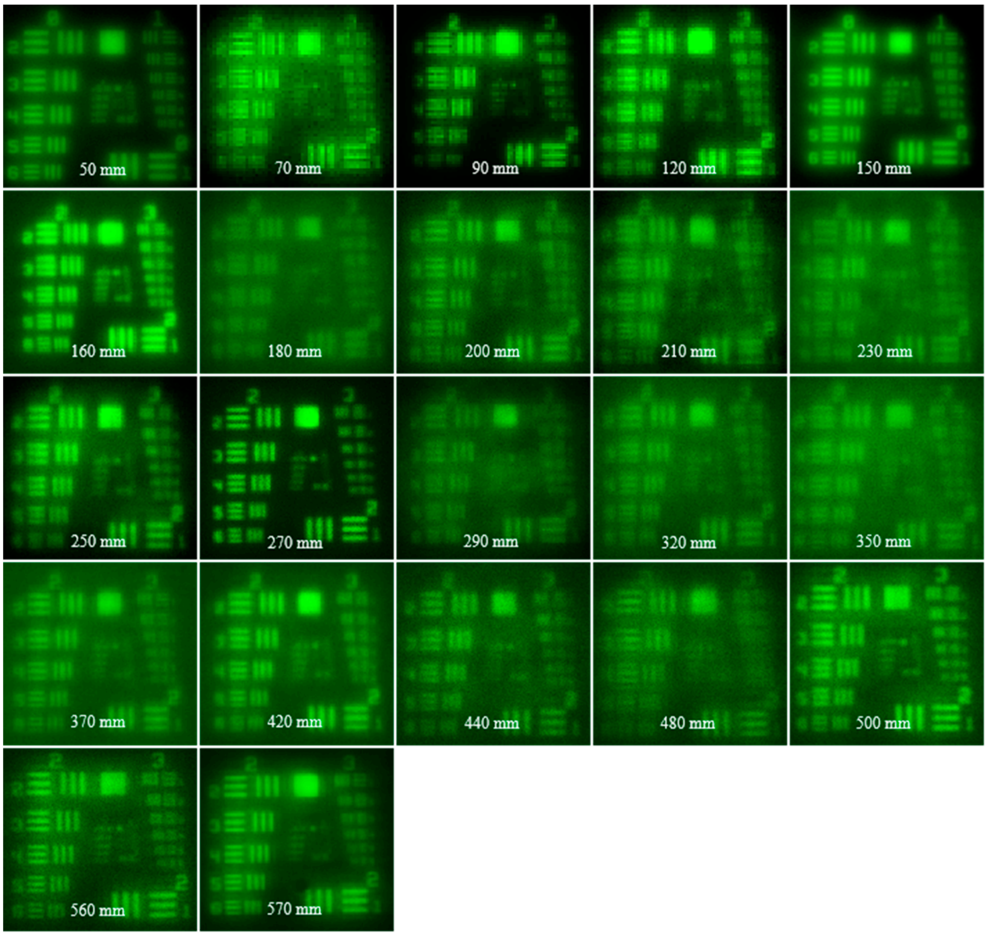

Figure S7. The resolution chart measured at the wavelength of 545 nm.

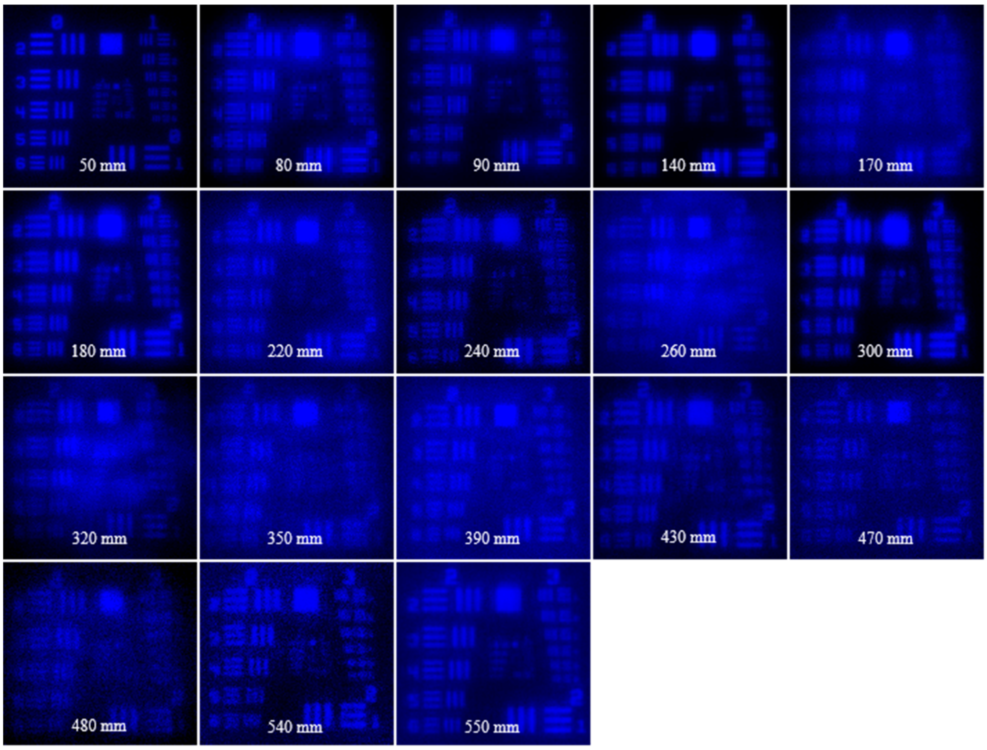

Figure S8. The resolution chart measured at the wavelength of 467 nm.

7. Experimental setup for imaging

The experimental setup for imaging with objects placed at various distance is shown in Fig. S9. The corresponding images captured by CCD sensor is shown in Fig. S6, Fig. S7 and Fig. S8.

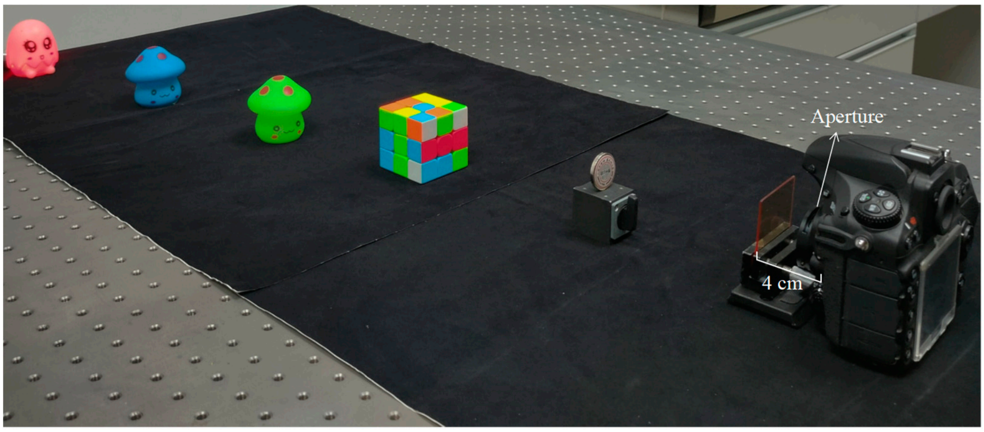

Figure S9. Experimental setup for imaging

References

1. L. L. Doskolovich; R. V. Skidanov; E. A. Bezus; S. V. Ganchevskaya, D. A. Bykov; N. L. Kazanskiy, “Design of diffractive lenses operating at several wavelengths,” Opt. Express, 2020, 28, 8, 11705-11720. DOI: 10.1364/OE.389458.
2. N Mohammad; M Meem; B Shen; P Wang; R Menon, “Broadband imaging with one planar diffractive lens,” Scientific reports, 2018, 8, 1, 1-6. DOI: 10.1038/s41598-018-21169-4.
3. S. Banerji; M. Meem; A. Majumder; B. Sensale-Rodriguez; R. Menon, “Extreme-depth-of-focus imaging with a flat lens,” Optica, 7, 3, 214-217. DOI: 10.1038/s41598-018-21169-4
4. N. Xu, Z. Kong, Q. Tan, and Y. Fu, “Multiring pure-phase binary optical elements to extend depth of focus,” Appl. Opt., 2018, 57, 32, 9643-9648. DOI: 10.1364/AO.57.009643.
5. C. Iemmi; J. Campos; J. C. Escalera; O. López-Coronado; R. Gimeno; M. J. Yzuel, “Depth of focus increase by multiplexing programmable diffractive lenses,” Opt. Express, 14, 22, 10207-10219. DOI: 10.1364/OE.14.010207.
